# Transcriptome analysis reveals differential immune related genes expression in *Ruditapes philippinarum* under hypoxia stress: potential HIF and NF-κB crosstalk in immune responses in clam

**DOI:** 10.1186/s12864-020-6734-6

**Published:** 2020-04-23

**Authors:** Hongtao Nie, Huamin Wang, Kunyin Jiang, Xiwu Yan

**Affiliations:** 1 College of Fisheries and Life Science, Dalian Ocean University, Dalian, 116023 China; 2 Engineering Research Center of Shellfish Culture and Breeding in Liaoning Province, Dalian Ocean University, Dalian, 116023 China

**Keywords:** *Ruditapes philippinarum*, Hypoxia stress, Transcriptomic analysis, HIF signaling pathway, Immune response, NF-κB signaling pathway

## Abstract

**Background:**

Hypoxia is an important environmental stressor in aquatic ecosystems, with increasingly impacts on global biodiversity. Benthic communities are the most sensitive parts of the coastal ecosystem to eutrophication and resulting hypoxia. As a filter-feeding organism living in the seafloor sediment, *Ruditapes philippinarum* represents an excellent “sentinel” species to assess the quality of marine environment. In order to gain insight into the molecular response and acclimatization mechanisms to hypoxia stress in marine invertebrates, we examined hypoxia-induced changes in immune-related gene expression and gene pathways involved in hypoxia regulation of *R. philippinarum*.

**Results:**

We investigated the response of the Manila clam *R. philippinarum* to hypoxia under experimental conditions and focused on the analysis of the differential expression patterns of specific genes associated with hypoxia response by RNA-seq and time course qPCR analysis. A total of 75 genes were captured significantly differentially expressed, and were categorized into antioxidant/oxidative stress response, chaperones/heat shock proteins, immune alteration, and cell proliferation/apoptosis. Fourteen hypoxia responsive genes were validated significantly up/down regulated at different time 0, 2, 5, and 8 d in gills of *R. philippinarum* in hypoxia challenged group. Functional enrichment analysis revealed the HIF signaling pathway and NF-κB signaling pathway play pivotal roles in hypoxia tolerance and resistance in *R. philippinarum*.

**Conclusion:**

The HIF signaling pathway and NF-κB signaling pathway play a critical role in hypoxia tolerance and resistance in Manila clam. The immune and defense related genes and pathways obtained here gain a fundamental understanding of the hypoxia stress in marine bivalves and provide important insights into the physiological acclimation, immune response and defense activity under hypoxia challenge. The reduced metabolism is a consequence of counterbalancing investments in immune defense against other physiological processes.

## Background

Hypoxia is considered to be one of the most important stressors to aquatic animals [1]. In recent years, hypoxia in marine habitat has drawn much more attention due to it is one of the ecological concerns in the world, especially the discharge of nutrient rich water and excessive anthropogenic input of organic matter into the sea [2]. Hypoxic layer often occurs near the sea floor during the summer in coastal areas, and leads to the mass mortality of the benthic animals [3, 4]. Notably, excessive nutrient enrichment and human activities may cause water eutrophication and result in hypoxic or anoxic bottom environments [5, 6]. Hypoxia may have remarkably impacts on aquatic animals at different levels. It has been reported that the behavioural, physiological and biochemical responses to hypoxia stress in several marine mollusca species [1, 7, 8].

Marine intertidal animals frequently encounter hypoxia or anoxia during low tide aerial exposure. Most sediment dwelling organisms are well adapted to the hypoxic aquatic realm and marine bivalves live in the intertidal region have to survive in the fluctuating environment at regular intervals. Indeed, intertidal bivalves are not passive to environmental changes and exhibit dynamic changes in their physiology, gene expression and metabolism to meet changes in their environment. When bivalves are challenged by hypoxia, a great number of physiological and molecular reactions are involved to deal with the low oxygen level stress [9]. One response of bivalves to aerial exposure or very low levels of hypoxia is to close their shells and regulate their internal environment. The respiratory activities usually slow down to adjust water flow and oxygen uptake, and reduce oxygen consumption with decreased energy expenditure [10]. In most marine invertebrates, the physiological adaptation to anaerobiosis caused by hypoxia or anoxia include maintaining reserves of energy store such as sugars or aspartate, and producing alternative end products to enhance ATP production [11]. Meanwhile, hypoxia may cause a slowdown of the process of reactive oxygen species (ROS) generation, and thus a decline in antioxidant enzymes activities [12], which could affect the immune and survival of shellfish species. So far, a number of studies have been conducted on the effects of hypoxia or anoxia on metabolic rate and modulation of enzyme activity in marine bivalves [13–15], as well as several research works in some shellfish species on molecular level [8, 16, 17].

Alkaline phosphatase (AKP) exerts a protective role in physiological function and immune defense such as detoxification of LPS and protection against endotoxin, an equally ubiquitous product of Gram-negative bacteria that may cause lethal complications after an infection with microorganisms [18, 19]. Succinate dehydrogenase (SDH) activity represents the physiological conditions of substrate oxidation and energy metabolism, along with mitochondrial function [20]. Lactate dehydrogenase (LDH) plays a critical role in maintaining aerobic metabolism by converting lactate, the major by-product of anaerobic glycolysis, to pyruvate via oxidation in the presence of its coenzyme nicotinamide adenine dinucleotide (NADH) [21]. Therefore, these enzymes AKP, SDH, and LDH can be used as biochemical and physiological markers (i.e. biomarkers) for the assessment of aquatic animal health conditions. Over the past decade, RNA sequencing (RNA-seq) has become an indispensable tool for transcriptome-wide analysis of differential gene expression and elucidation of the mechanisms underlying suites of ecological processes [22]. The differentially expressed genes with oxygen depletion stress and transcriptional responses to hypoxia were reported in the oyster *Crassostrea gigas* [8], mussel *Mytilus galloprovincialis* [16], and abalone *Haliotis diversicolor* [17].

The Manila clam, *Ruditapes philippinarum*, is an economically and scientifically important marine bivalve species, which has a wide geographic distribution from Europe to Asia [23]. Manila clam is one of the major aquaculture species in the world and the production of *R. philippinarum* reached over 4.0 million tons, equivalent to 3.7 million USD, in 2015 [24]. In recent years, however, this species faces greater risk of exposure to hypoxia as eutrophication worsens throughout its coastal habitats. As a filter-feeding organism living in the seafloor sediment, *R. philippinarum* represents an excellent “sentinel” species to assess the quality of marine environment [25]. In the past decades, hypoxia has become a serious issue in coastal environmental conservation, since some of the clams die because of the hypoxic water [26, 27]. Hence, the tolerance of the bivalves for hypoxia and/or anoxic conditions has been investigated by a number of researchers [1, 26, 27]. More recently, the effects of hypoxia on survival, behavior, metabolism and cellular and tissue damage of *R. philippinarum* were conducted, suggesting that severe hypoxia significantly affects the physiology of *R. philippinarum* [28, 29]. Until now, however, little effort has been put towards elucidating the molecular mechanisms combined with physiological and biochemical response of clams to the hypoxia resistance and acclimations. The underlying mechanisms of molecular alterations and responses to hypoxia stress in *R. philippinarum* remains largely unknown.

In this study, RNA-seq approach was adopted to investigate the transcriptome profiles of the gills from *R. philippinarum* under hypoxia and normoxia conditions. This work aimed to identify the differentially expressed genes and their expression patterns under low oxygen challenge to better understand the transcriptomic regulation in response to hypoxia stress and to investigate hypoxia-induced changes in immune-related gene expression and gene pathways involved in hypoxia regulation of *R. philippinarum*. Meanwhile, the responses of the energy metabolism and immune related enzymes (SDH, LDH and AKP) were investigated in *R. philippinarum* under hypoxia challenge at experimental conditions. These results provide new insights into understanding of the integrative regulation mechanism at the physiological, biochemical and the molecular level involved in hypoxia tolerance and resistance in *R. philippinarum*.

## Results

### Transcriptome sequencing, assembly and GO analysis

**Fig. 1:**
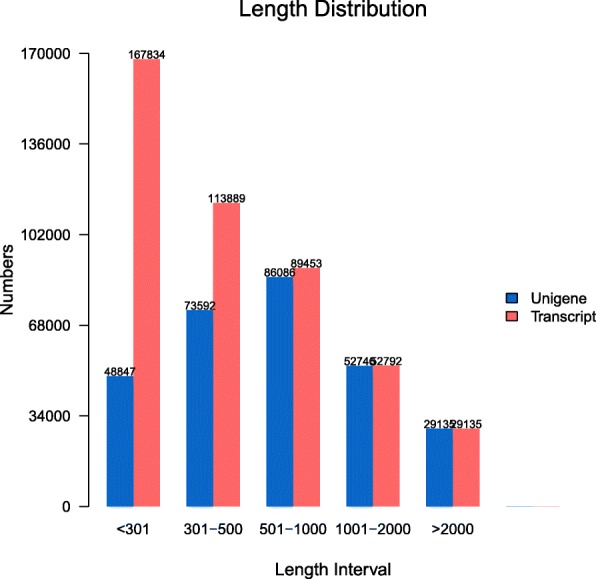
Sequence length distribution of transcripts and unigenes assembled from Illumina reads of the transcriptome of *Ruditapes philippinarum*. The x-axis indicates the lengths of the transcripts and unigenes, and the y-axis indicates the number of transcripts and unigenes in each size category

**Fig. 2:**
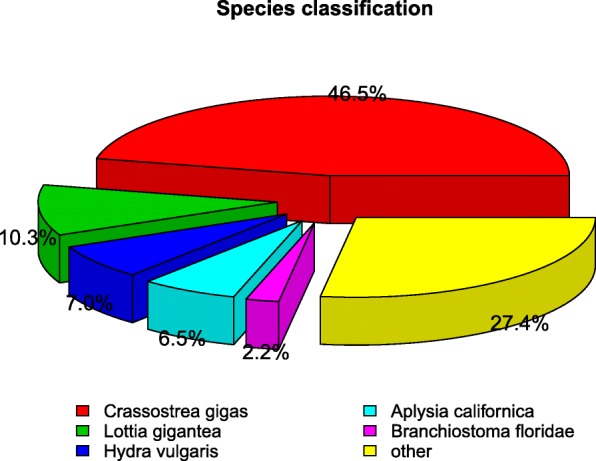
Species distribution of sequences matched to the Nr database

To investigate the effects of hypoxia on their molecular pathways and stress responsive genes involved in this processes. Six RNA-seq libraries were constructed using gills from three clams under hypoxia treatment (H1, H2 and H3) and three clams in normoxia control (N1, N2 and N3) to generate a reference transcriptome of *R. philippinarum*. Library sequencing yielded a total of 297,734,780 raw reads with a mean of 125 bp from six libraries. After filter of low-quality reads, 283,487,670 (95.2%) high quality reads were retained and de novo assembled. Clean reads obtained for H1, H2, H3, N1, N2, and N3 have been submitted to the SRA database in NCBI (accession numbers: PRJNA478917). A total of 290,406 unigenes were obtained with an average length of 935 bp (Minimum length: 201 bp, Maximum length: 46,346 bp, N50 length: 1389 bp) (Table 1). The length distribution of the unigenes is shown in Fig. 1. In the distribution of sequences annotated in Nr, 46.5% unigenes have similarities with *Crassostrea gigas*, followed by *Lottia gigantea* (10.3%), *Hydra vulgaris* (7.0%), *Aplysia californica* (6.5%), *Branchiostoma floridae* (2.2%), and others (27.4%) (Fig. 2).

**Table 1:** Summary statistics of *Ruditapes philippinarum* transcriptome assembly using Trinity software

Unigene	Min. Length (bp)	Mean Length (bp)	Max. Length (bp)	Median Length (bp)	N50	N90	Total Length (bp)
Number	201	935	46,346	587	1389	408	271,479,222

**Fig. 3:**
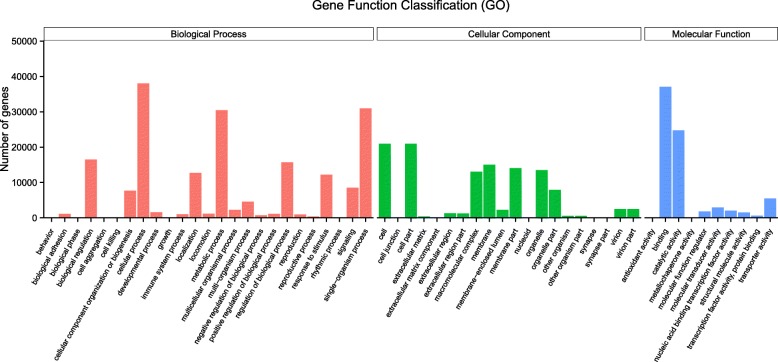
GO (Gene Ontology) categorization (biological process, cellular component, and molecular function) of the unigenes in the gill transcriptome of *R. philippinarum*. Each annotated sequence is assigned at least one GO term

All the unigenes were enriched in the Kyoto Encyclopedia of Genes and Genomes (KEGG) pathways and Gene Ontology (GO) terms (Table 2). Differentially expressed genes involved in peroxisome, mitochondrial envelope, and transferring phosphorus-containing groups were identified in the cellular components (Additional file 1). For the molecular functions, genes related to catalytic activity, binding, oxidoreductase activity, peptidase activity, peroxiredoxin activity and transferase activity highly represented among the molecular functions (Fig. 3, Additional file 2). In this study, several hypoxia regulated functions such as regulation of apoptotic process, immune system process, antioxidant activity, immune response, tumor necrosis factor receptor binding, peroxidase activity, and oxidoreductase activity were shown in Table 3. Decrease in defense response and response to stress activity might be related to regulation of response to oxidative stress, cell surface receptor signaling pathway, and reactive oxygen species (Additional file 1). Moreover, enzymes involved in immune response, antioxidant activity, peroxidase activity, oxidoreductase activity, peroxiredoxin activity, cytokine receptor binding and regulation of apoptotic process were enriched under hypoxia conditions (Table 3), indicating that *R. philippinarum* might acclimated to hypoxia through physiological, immune and defense response to stress. Additionally, a number of genes were involved in important categories, including cellular nitrogen compound biosynthetic process, organonitrogen compound catabolic process, cellular protein metabolic process, and macromolecular complex were also identified (Additional file 1), which may play potential roles in the hypoxia tolerance of *R. philippinarum*.

**Table 2:** Summary statistics of functional annotation of *R. philippinarum* transcriptome

Category	All sequences	300-999 bp	> = 1000 bp
Total number of unigene	148,593	64,105	26,610
Unigene matches against Nr and Swiss-Prot	20,955	5787	12,873
Unigene matches against GO	32,370	11,094	15,442
Unigene matches against KEGG	10,434	2985	6253

**Table 3:** GO classification of the differentially expressed genes from *R. philippinarum* under hypoxia stress

Term	Comparison	Type	Gene	*p*-Value
immune system process (GO:0002376)	H1 vs N1	BP	71	0.000218
antioxidant activity (GO:0016209)	H1 vs N1	MF	20	0.000309
immune response (GO:0006955)	H1 vs N1	BP	60	0.000311
tumor necrosis factor receptor binding (GO:0005164)	H1 vs N1	MF	20	0.000474
peroxidase activity (GO:0004601)	H1 vs N1	MF	7	0.003901
regulation of apoptotic process (GO:0042981)	H1 vs N1	BP	58	0.004872
oxidoreductase activity (GO:0016491)	H2 vs N2	MF	278	3.24E-07
antioxidant activity (GO:0016209)	H2 vs N2	MF	23	2.05E-05
peroxiredoxin activity (GO:0051920)	H2 vs N2	MF	7	0.000251
immune system process (GO:0002376)	H3 vs N3	MF	61	0.000900
antioxidant activity (GO:0016209)	H3 vs N3	MF	19	0.000159
immune response (GO:0006955)	H3 vs N3	BP	51	0.001554
tumor necrosis factor receptor binding (GO:0005164)	H3 vs N3	MF	21	2.92E-05
cytokine receptor binding (GO:0005126)	H3 vs N3	MF	34	4.99E-05
defense response (GO:0006952)	H vs N	BP	5	0.000556
response to stress (GO:0006950)	H vs N	BP	7	0.007614
oxidoreductase activity (GO:0016491)	H vs N	MF	8	0.005337
peroxiredoxin activity (GO:0051920)	H vs N	MF	1	0.017815

### KOG analysis and KEGG classification

**Fig. 4:**
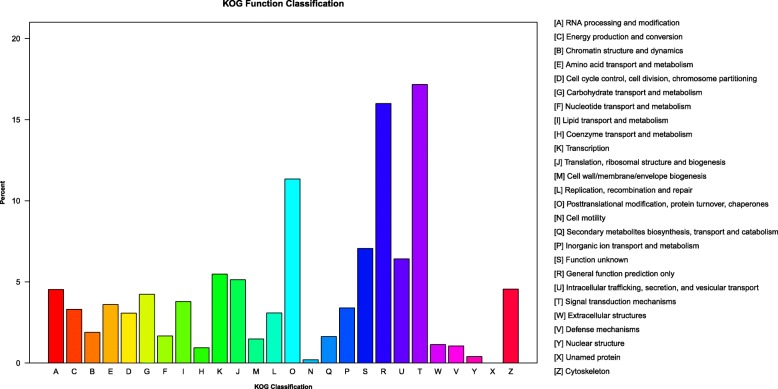
KOG (euKaryotic Ortholog Groups) classifications of putative proteins in the gill transcriptome of *R. philippinarum*

Eukaryotic Orthologous Groups (KOG) analysis was performed to classify all unigenes into different functional categories (Fig. 4). Annotated genes in these categories are probably related to energy production and conversion, signaling transduction, intracellular free amino acid transport and metabolism, cell signaling pathways, and regulation of ionic content and cell volume. A total of 22,862 unigenes were classified into 231 different pathways through KEGG pathway analysis, such as metabolism, cellular processes, and organismal systems. The metabolic pathways with most unigenes divide into 11 groups, including carbohydrate metabolism, energy metabolism, lipid metabolism, amino acid metabolism, glycan biosynthesis and metabolism, and so on (Table 4). All DGEs from the comparison of Hypoxia and control group were mapped to KEGG for identification of the biological pathways in response to hypoxia. It is indicated that immune-related pathways were enriched, such as NF-κB signaling pathway, Toll-like receptor signaling pathway, complement and coagulation cascades, chemokine signaling pathway, and PI3K-Akt signaling pathway (Additional file 3).

**Table 4:** Kyoto Encyclopedia of Genes and Genomes (KEGG) pathway mapping for *R. philippinarum*

KEGG Pathways	Sub-Pathways	Number of Unigenes
Cellular Processes	Cell growth and death	966
	Cell motility	451
	Cellular commiunity	1340
	Transport and catabolism	1802
	Membrane transport	118
	Signal transduction	3410
	Signaling molecules and interaction	680
Genetic Information Processing	Folding, sorting and degradation	1470
	Replication and repair	425
	Transcription	609
	Translation	1346
Metabolism	Amino acid metabolism	839
	Biosynthesis of other secondary metabolites	39
	Carbohydrate metabolism	960
	Energy metabolism	324
	Glycan biosynthesis and metabolism	598
	Lipid metabolism	964
	Metabolism of cofactors and vitamins	401
	Metabolism of other amino acids	393
	Metabolism of terpenoids and polyketides	71
	Nucleotide metabolism	596
	Xenobiotics biodegradation and metabolism	202
Organismal Systems	Circulatory system	621
	Development	614
	Digestive system	1014
	Endocrine system	1815
	Environmental adaptation	366
	Excretory system	375
	Immune system	1296
	Nervous system	1097
	Sensory system	420

### Hypoxia responsive genes and molecular pathways in Manila clam

**Fig. 5:**
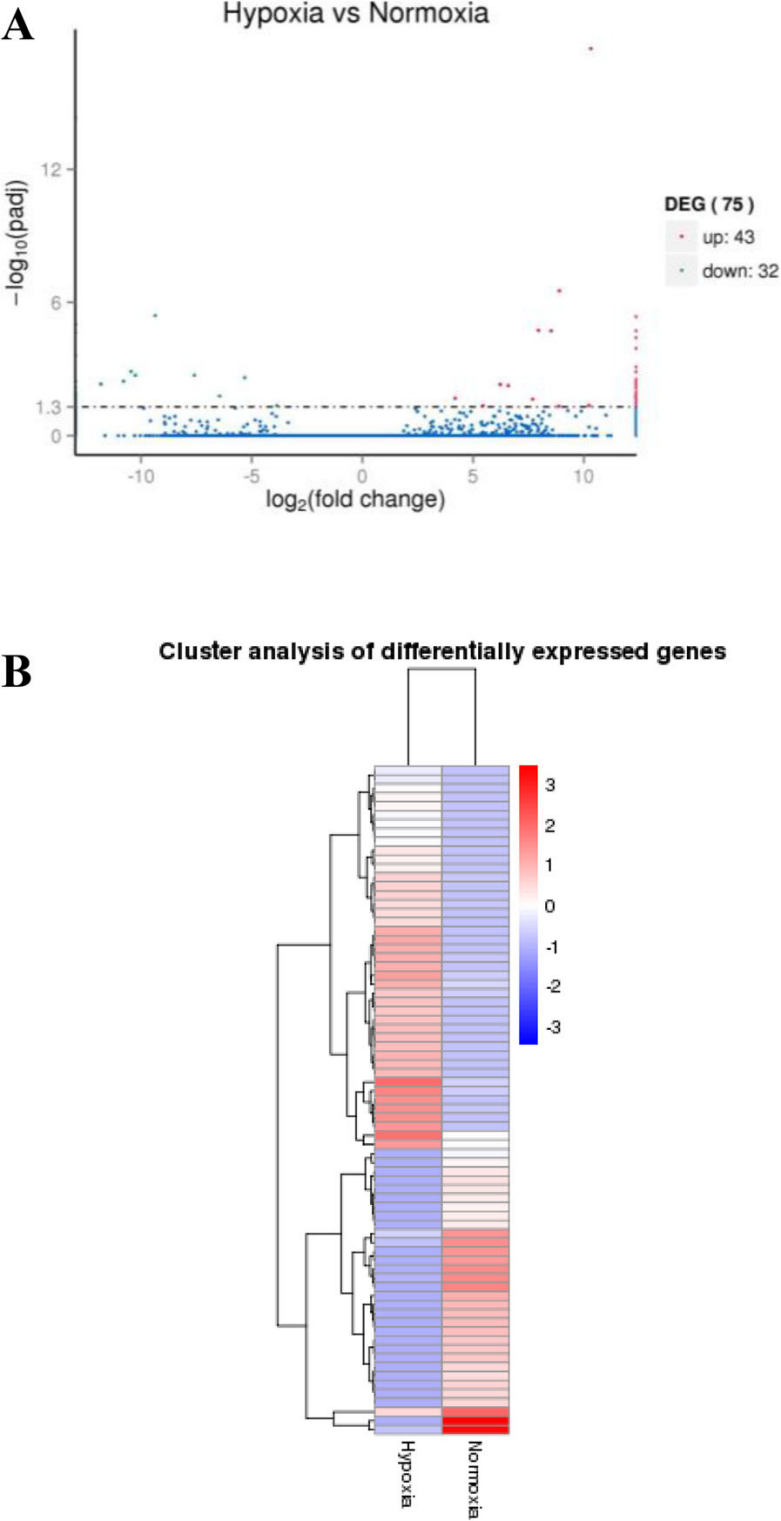
Volcano plot of differentially expressed genes (DEGs) from the transcriptomes of H vs N in *R. philippinarum* (**a**), Heatmap of Cluster analysis of DEGs from the transcriptomes of Hypoxia (H) vs Normoxia (N) in *R. philippinarum* (**b**)

In this study, 148,358,474 and 135,129,196 qualified Illumina read pairs were obtained from the hypoxia challenged group (H1, H2, and H3) and normoxia control group (N1, N2, and N3) of *R. philippinarum*. RNA-seq analysis revealed that only 75 unigenes were differentially expressed genes (DEGs) under hypoxia challenge (qvalue< 0.005 and |log2(foldchange)| > 1). Among these, 32 DEGs were down-regulated and 43 DEGs were up-regulated (Fig. 5a, Additional file 4). Heatmap of cluster analysis of DEGs from the transcriptomes of hypoxia and normoxia in *R. philippinarum* was shown in Fig. 5b.

**Fig. 6:**
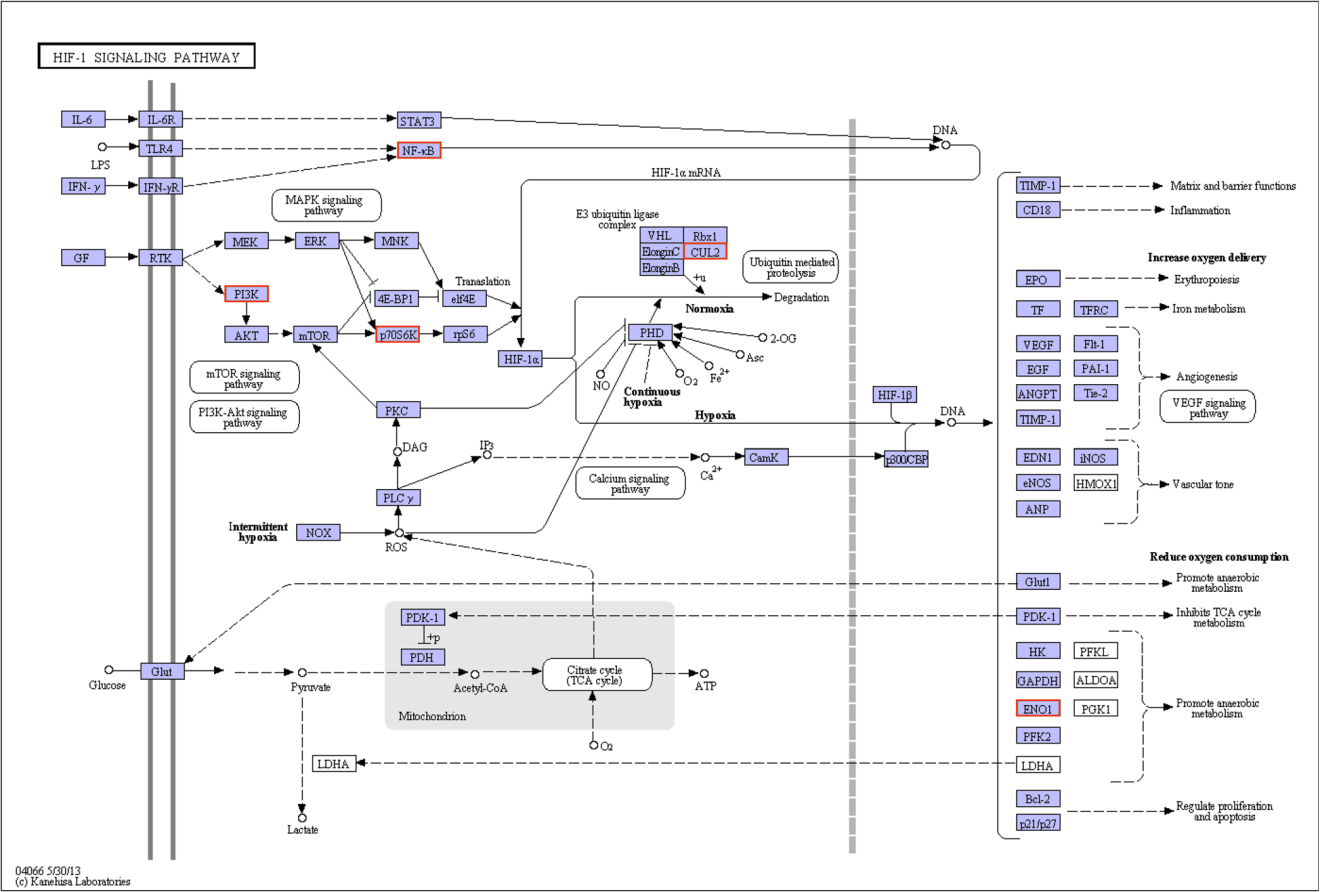
The HIF signaling pathway and upregulated DEGs in HIF pathway from KEGG enrichment analysis of *R. philippinarum* under hypoxia challenge. The red box indicates the upregulated genes in HIF pathway

A number of hypoxia responsive pathways, including the HIF-1 signaling pathway, NF-kappa B signaling pathway, AMPK signaling pathway, Apoptosis, Jak-STAT signaling pathway, MAPK signaling pathway, PI3K-Akt signaling pathway, Rap1 signaling pathway, Ras signaling pathway, TNF signaling pathway, PPAR signaling pathway, Notch signaling pathway, Wnt signaling pathway, Calcium signaling pathway, cAMP and cGMP/PKG signaling pathway were identified in this study (Additional file 1). In HIF-1 signaling pathway, the NF-κB, PI3K and p70S6K are upregulated, which could increase the HIF-1α mRNA or HIF-1α (Fig. 6). Consequently, the downstream gene of the HIF signaling pathway, enolase (ENO1), is significantly upregulated in the hypoxia challenged clams, which promote anaerobic metabolism and causing reduced oxygen consumption.

**Fig. 7:**
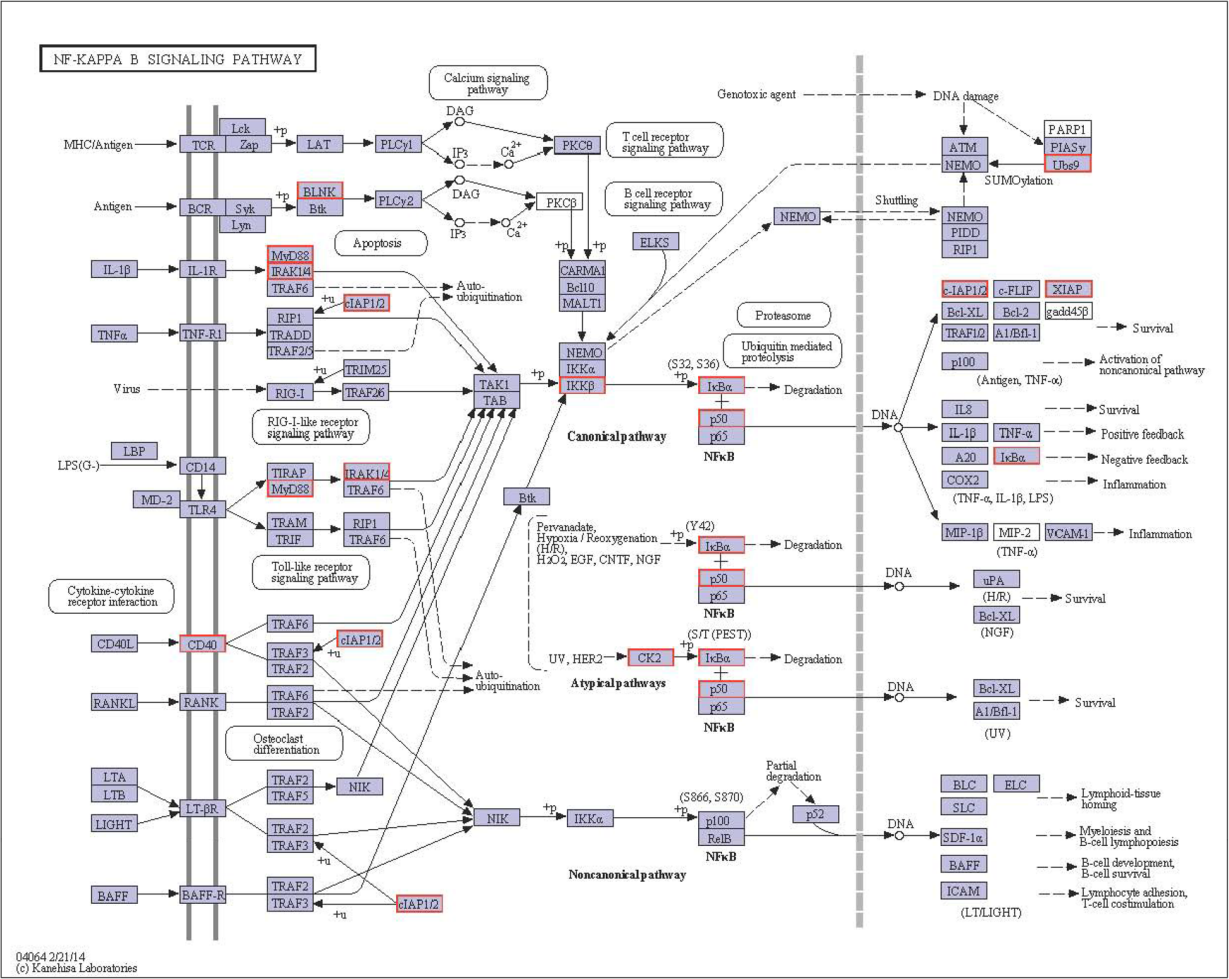
The NF-κB signaling pathway and upregulated DEGs in NF-κB signaling pathway from KEGG enrichment analysis of *R. philippinarum* under hypoxia challenge. The red box indicates the upregulated genes in NF-κB signaling pathway

Nuclear factor kappa B (NF-κB) activation starts with the phosphorylation, ubiquitination and subsequent proteosomal degradation of IκB (Fig. 7). NF-κB complex could enter the nucleus with the IκB degradation, and then it turn on the expression of specific genes with DNA-binding sites for NF-κB. Genes activated by NF-κB result in physiological response, for instance, an immune/inflammatory response, cellular proliferation and survival response. As shown in Fig. 7, the NF-κB turns on expression of its own repressor IκBα, and then the IκBα inhibits NF-κB, and thus forms a feedback loop, which regulates the activity of NF-κB [30]. These pathways play potential roles in the signal transduction, intracellular transduction, and sensing of stress signals, endocrine system and immune response of *R. philippinarum* under hypoxia stress.

### The effects of hypoxia on SDH, LDH and AKP enzyme activity

**Fig. 8:**
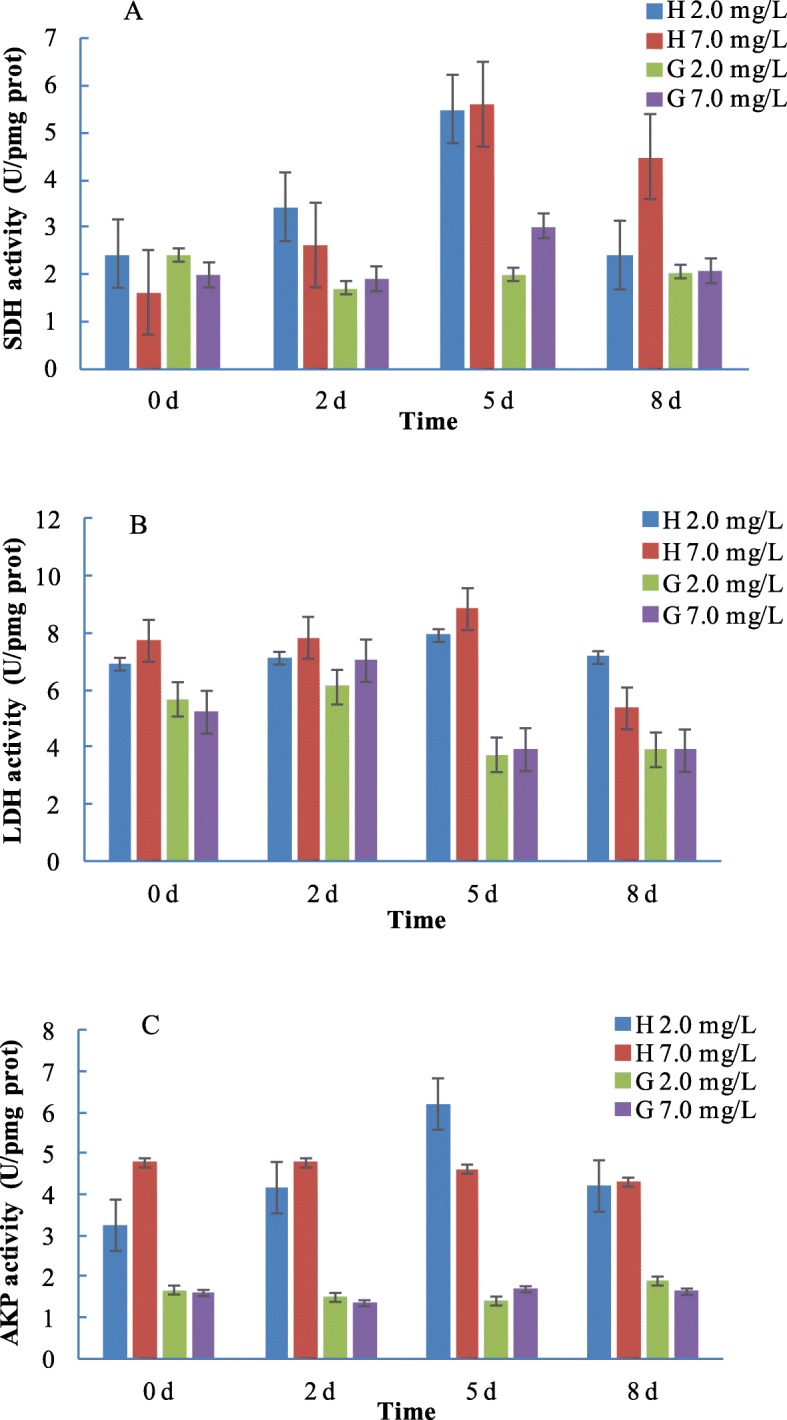
The enzymes activities of SDH, LDH and AKP at different exposure duration (0, 2, 5, and 8 d) of clams in normoxia control groups (7.0 mg/L) and hypoxia challenged groups (2.0 mg/L). H: hepatopancreas, G: gills. * indicate significant different at *P* < 0.05

As shown in Fig. 8a&b, the succinate dehydrogenase (SDH) and the lactate dehydrogenase (LDH) activity in hepatopancreas of *R. philippinarum* increased first and reach a peak at 5 d, and then decreased at 8 d. In hypoxia group, the activity of SDH in hepatopancreas at 8 d significantly lower than that in control group, while the activity of LDH in hepatopancreas at 8 d was higher in hypoxia group compared to that in control group, which may be caused by clams switch to anaerobic metabolism at 8 d hypoxia stress. The activity of alkaline phosphatase (AKP) in hepatopancreas was significantly increased to a maximum at 5 d and then decreased at 8 d under hypoxia challenge, while almost no change of the AKP activity in control group was observed (Fig. 8c). In the gills, however, no significant change in AKP activity occurs between hypoxia challenge and control group from 0 to 8 d (8 days), and an organ/tissue-specific response was observed, suggesting AKP might be involved in defense response and immune function in hepatopancreas of *R. philippinarum*.

### Time-course of transcripts detected in hypoxia challenged clams

**Fig. 9:**
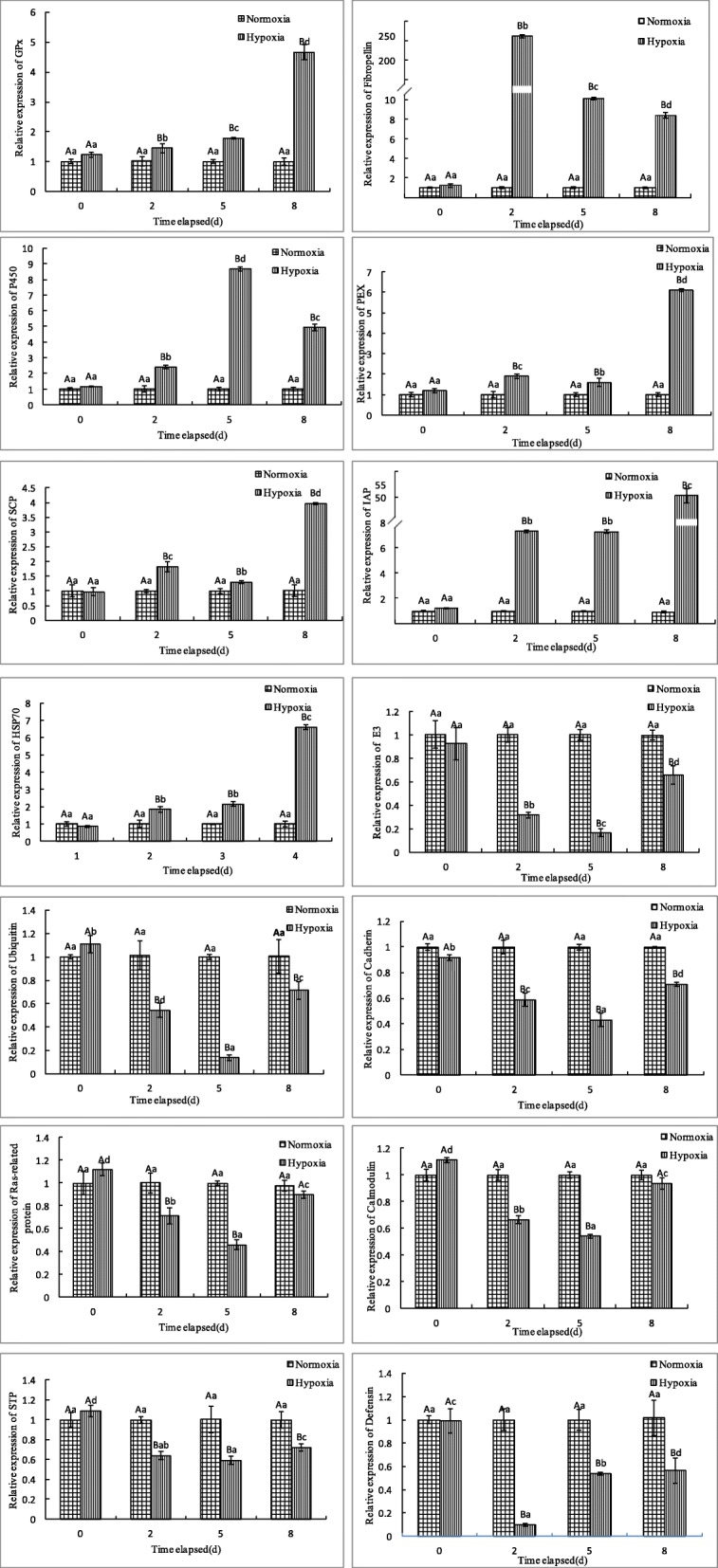
Validation of RNA-Seq results using RT–qPCR. The transcript expression levels of the selected genes were normalized to that of the β-actin gene

To corroborate the DEGs identified in the RNA-seq analysis, hypoxia responsive genes were selected from the DEGs for quantitative real-time PCR (qPCR) validation from the genes of interest based on their functions. The expressions values of 14 genes were analyzed in two tissues of Manila clam at different times of hypoxia stress. The expressions of 14 hypoxia responsive genes were significantly different between hypoxia challenged group and control group at different time 2, 5, and 8 d under hypoxia stress (Fig. 9). Under hypoxia conditions, the Cytochrome P450, HSP70, Peroxisomal membrane protein (PEX), Sterol carrier protein (SCP), Glutathione peroxidase (GPx), Fibropellin-1, and Inhibitor of apoptosis protein (IAP) are expressed at a significant higher level at 2, 5, and 8 d hypoxia exposure than in control groups. While the Cadherin, Calmodulin, Defensin, Ubiquitin-conjugating enzyme E2, Serine/threonine-protein phosphatase, E3 ubiquitin-protein ligase and Ras-related proteins are expressed at a lower level at 2, 5, and 8 d hypoxia exposure compared to the control groups (Fig. 9).

As shown in Fig. 9, the expression of Fibropellin-1 increased 248.9-fold higher levels in 2 d post hypoxia exposure compared to normoxia control group. HSP70 are significantly up-regulated in *R. philippinarum* under hypoxia stress with 6.6-fold up-regulation at 8 d compared with control group. The expression value of IAP increased significantly from 7.3 at 2 d to 50.5 at 8 d under hypoxia stress. The expression of GPx and PEX increased 4.66 and 6.12-fold higher levels in 8 d post hypoxia exposure compared to normoxia control group. On the other hand, the expression of Cadherin and Calmodulin was decreased to 0.59 and 0.66-fold lower levels at 2 d compared with control group. The expression value of E3 ubiquitin-protein ligase and Ubiquitin-conjugating enzyme E2 decreased significantly from 0.32 and 0.54 at 2 d to 0.17 and 0.14 at 5 d under hypoxia stress, respectively. The expression value of Defensin was significantly decreased over 10-fold down-regulation at 2 d hypoxia treatment compared with control group. Overall, the qPCR validation results of 7 up-regulated genes and 7 down-regulated genes were consistent with the RNA-seq analysis.

## Discussion

Hypoxia activates a variety of complex pathways at both the cellular and organism level, with the ultimate aim of reinstating oxygen homoeostasis. In the past decades, physiological and biochemical responses to hypoxia have been studied in several marine bivalve species [26, 28, 31, 32]. Adenosine-5′-triphosphate (ATP), the major energy source for cells in the body, is predominantly supplied by a series of metabolic pathways including glycolysis, the citric acid cycle, and the electron transport chain [33]. Among these, glycolysis occurs (with variations) in nearly all organisms, both aerobic and anaerobic. The wide occurrence of glycolysis indicates that it is one of the most important energy metabolic pathways [34]. It provides not only high-energy compounds like ATP, but also pyruvate, which can be used in the citric acid cycle to generate more ATP, NADH, and FADH_2_ [35–37]. In this study, the enzymatic assays of LDH indicated that the hepatopancreas of *R. philippinarum* switch to anaerobic metabolism at 8 d under hypoxia at 15 °C, while the SDH activity is likely to be related to mitochondrial aerobic energy production, suggesting that aerobic and anaerobic metabolism were both existed in *R. philippinarum*. When Manila clams are under hypoxia, their shell valves are frequently closed, likely undergoing low oxygen consumption and reduced metabolisms [38]. The activity of LDH is closely related to cell metabolism, and its activity is often used as an indicator to evaluate the level of anaerobic metabolism [39]. As shown in Fig. 8a&b, the level of dissolved oxygen in the water is insufficient with the hypoxia exposure time increased, and the clams may depend on anaerobic respiration to supply the metabolic energy, which is strongly reducing their energy requirement, so the activity of SDH and LDH decreases with prolonged hypoxia challenge. The different changes in the activities of key enzymes involved in glycolysis may indicate a diverse strategy of shellfish under hypoxia [29].

The hypoxia condition is a challenging situation because the energy production is drastically decreased in anaerobic metabolic pathways compared to aerobic energy provision. The decrease in metabolic rate at hypoxic environment is mainly caused by two mechanisms, including the depression of ATP-requiring processes and the inhibition of ATP-generating pathway. The reduction of the metabolic rate under hypoxia challenge provides benefits for facultative anaerobes. The glycogen stores of *Mytilus edulis* would only be enough for 3 days to provide ATP by anaerobic fermentation. However, mussels are able to sustain their life at anoxic conditions for weeks by reducing energy requirement [40]. The Manila clam exhibited strong tolerance to hypoxia as the 20-day LC_50_ for dissolved oxygen (DO) was estimated to be 0.57 mg L^− 1^, and the LT_50_ at 0.5 mg L^− 1^ DO was 422 h [29]. In this study, AKP activity in hepatopancreas was significantly increased at 5 d in response to hypoxia stress, indicating that the hepatopancreas is an important organ that involved in the immune response and hypoxia regulation in *R. philippinarum*. In the gills, however, no significant change in AKP activity observed between hypoxia challenge and control groups during 8 days, suggesting a tissue-specific response exist in *R. philippinarum*, which was also reported in *C. gigas* [1].

Previous studies explored how environmental changes are transduced into coordinated changes in physiological processes by examining gene expression cycles using microarray in intertidal bivalves [41–46]. More recently, transciptomic profiling is widely employed in elucidating the genetic basis of molecular response to different environmental factors in marine bivalve [47, 48]. In this study, a transcriptomic approach was firstly employed to investigate the gene expression patterns of the Manila clam under hypoxic challenge. Hypoxia responsive genes associated with stress response, substance and energy metabolism, and immune response were corroborated by qPCR. In this study, all of the 14 DEGs detected by qPCR were consistent with the RNA-seq results. The expression profiles of these genes were characterized at different hypoxia exposure duration (0, 2, 5, and 8 d). These genes reflect different aspects of the stress adaptation and molecular mechanisms of immune response to hypoxia exposure. The KEGG analyses of DEGs show that molecular pathways related to TNF signaling, NOD-like receptor signaling, RIG-I-like receptor signaling, TGF-beta signaling AMPK signaling, NF-kappa B signaling, cAMP signaling pathway, and apoptosis are highly enriched, implicating these processes are important for physiological adaptation and immune response to hypoxia stress in *R. philippinarum*. Manila clam may have evolved various sophisticated signaling pathways to sense their immediate environment and orchestrate appropriate transcriptional responses that mediate adaptation in benthic environment.

Aerobic organisms have the antioxidant mechanisms to prevent the oxidative damage and protect them against oxidant stress. This biological process involve in a number of cellular molecules and antioxidant enzymes [43]. In the last decade, however, most studies in marine bivalves investigated the activities of the antioxidant enzymes, but only a few studies reported both the biochemical responses and molecular actors governing the antioxidant following hypoxia [49, 50]. In this study, we observed a rapid regulation of a set of genes involved in the molecular responses to oxidative stress. The cytochrome P450, peroxisomal membrane protein (PEX), and Glutathione peroxidase (GPx) were both upregulated under hypoxia challenge. Peroxidase, an important class of antioxidation enzymes, is responsible for the defense response to oxidative stress and degradation of ROS [51]. The expression of GPx mRNA increased under hypoxia challenge to protect cells from ROS that can be formed upon reoxygenation, which has been reported in *C. gigas* [1, 8]. Stress-induced immune changes have been reported in many marine invertebrates, including oyster [52, 53] and mussel [45, 54]. The innate immunity is the mainly immunological defense mechanism in invertebrate metazoan [55]. In the present study, the expression of several critical genes were significantly up-regulated (IAP and HSP70) and down-regulated (Defensin) under the hypoxia challenge, demonstrating that these genes are potentially involved in defense and immune response. In addition, focal adhesion, NF-kappa B signaling pathway, apoptosis, TNF signaling pathway were enriched by KEGG analysis, suggesting that these pathways play significant roles in the immune response and defense mechanisms against hypoxia stress.

Hypoxia inducible factors (HIFs) are a family of highly conserved transcription factors that act as main regulators of oxygen homeostasis and the adaptive response to hypoxia [56]. NF-κB signaling pathway has been proved to be involved in innate immune response to bacterial infection and hypoxia stress in molluscan shellfish, including *R.philippinarum* [57], *Meretrix meretrix* [58], *C. gigas* [59], and *H. diversicolor* [17]. It is indicated that genes associated with NF-κB signaling pathway participated in the immunomodulation process to respond to hypoxia stress [17]. Therefore, we focus on the HIF-1 and NF-kappaB signaling pathways in the present study. Nuclear factor kappa B (NF-κB) is a transcription factor regulates the expression of cytokines, effector enzymes and apoptosis inhibitors in response to extracellular signals. NF-κB signaling plays a critical role in immune defense, stress responses and inflammation. NF-κB comprises RelA (p65), RelB, c-Rel, NF-κB1 (p50) and NF-κB2 (p52). NF-κB can stimulate transcription of its target genes in a very quick fashion, as it exists freely in the cytoplasm, albeit inhibited by IκB proteins. In the current study, NF-kB is upregulated, which could increase the HIF-1α mRNA level, as well as the PI3K and p70S6K are upregulated in PI3K-Akt signaling pathway.

Enolase is a hypoxic stress protein, which may contribute to hypoxic tolerance by increasing anaerobic metabolism [60]. As one the downstream genes of the HIF signaling pathway, the enolase (ENO1) is significantly upregulated in the hypoxia challenged clams, which promote anaerobic metabolism and causing reduced oxygen consumption. Concomitantly, hypoxia induced NFκB activity is preceded by temporally sequential IKK activation, IκB phosphorylation, and IκB degradation, indicating that hypoxia activates NFκB through increased IKK activity (Fig. 7). The IKK-dependence of hypoxia-induced NFκB activity is consistent with most other stimuli of this pathway [61]. The role of IKK in oncogenesis and inflammation is well established, but there is emerging evidence that IKK may have anti-inflammatory functions [62, 63]. In this study, we also observed an increase in IKKβ in hypoxia (Fig. 7). The observation that IKKβ are hypoxia-sensitive, suggests the possibility of a molecular mechanism where a p50- mediated response could be resolved over a course of hypoxia [61]. Therefore, NF-κB plays important roles in regulating genes responsible for both the immune response and hypoxia stress resistance.

## Conclusions

The physiological, biochemical and molecular responses of *R. philippinarum* under hypoxia stress were firstly investigated in the present work. This study provides new insights and comprehensive understanding of the molecular mechanism for hypoxic tolerance and resistance in *R. philippinarum*. The significant differentially expressed genes in *R. philippinarum* under hypoxia stress are involved in the antioxidant/oxidative stress response, chaperones/heat shock proteins, immune alteration, and cell proliferation/apoptosis. The reduced metabolism might be the consequence of counterbalancing investments in immune defense against other physiological processes. We speculate that it is likely to be an adaptive strategy for Manila clam to survive in low oxygen sediment and live in buried life. This study provides useful information for further study of the critical molecular molecules regulating the hypoxia tolerance and gain new insights on mechanisms of resistance to hypoxia stress in marine bivalves.

## Methods

### Clam acclimation, hypoxia challenge and sampling

Manila clams were collected from Zhuanghe, Dalian, China. After being transported from the field to the laboratory, the clams were cleaned to remove any fouling and were acclimated for 1 week in aerated 70 L plastic tanks, containing water at 15 ± 0.5 °C with salinity of 32 ppt. Other water quality parameters were measured during the experiment (pH: 8.0 ± 0.2, dissolved oxygen: 7.0 ± 0.5 mg/L). All the clams were fed with Spirulina powder daily for 1 week before the hypoxia challenge, and water was exchanged once per day to discharge waste products from marine invertebrates. Manila clam is not an endangered or protected species, so no specific permits were required for this study.

For the hypoxia challenge, clams were placed in another closed tank in which O_2_ concentration was controlled by monitor through nitrogen injection. A feedback was provided to the monitor by an O_2_ sensing probe (YSI ProPlus, USA) placed in the tank and nitrogen flow was adjusted in order to maintain O_2_ level to a set-value (2.0 mg/L). A total of 120 clams with similar size were divided into six groups in separate tanks. Hypoxia challenge group (H1, H2 and H3) was kept at low oxygen level (2.0 mg/L), and normoxia group (N1, N2 and N3) was at normal oxygen level (7.0 mg/L). The temperature and salinity of seawater were at 15 °C and 32 ppt, respectively. The gill sample from Manila clams in hypoxia challenge groups and control groups after different time were sampled, respectively, and frozen in liquid nitrogen and stored at − 80 °C until RNA extraction. Each sample was performed in triplicate. Meanwhile, the gills and digestive gland of Manila clams were sampled at 0, 2, 5, 8 d with three clams per tanks for each of the hypoxia challenge groups and control groups (three biological replicates with three technical replicates).

### RNA extraction and tissue samples preparation

The gills from three Manila clams in hypoxia challenge groups and control groups after 24 h were sampled, respectively, and the RNA was extracted for transcriptome sequencing. Total RNA was extracted from 30 mg gill sample from each individual using RNAprep pure Tissue Kit (TianGene, Beijing, China), according to the manufacturer’s protocol. RAN degradation and contamination was monitored on 1% agarose gels. RNA concentration and integrity were measured using a Qubit RNA Assay Kit in Qubit2.0 Flurometer (Life Technologies, CA, USA) and RNA Nano 6000 Assay Kit of the Agilent Bioanalyzer 2100 system (Agilent Technologies, CA, USA).

About 100 mg sample of gills and digestive glands were taken from each randomly chosen individual from each tank in the hypoxia challenge groups and control groups. Glass homogenizers were used to homogenize this tissue on ice for 2 min. The frozen samples were treated with 5 volumes of 0.05 M PBS buffer (pH 7.5). The homogenates were centrifuged for 8 min at 3500×*g*, and the supernatant was collected and assayed for enzyme activities. All samples were tested using analysis kits manufactured by Nanjing Jiancheng Bioengineering Institute (Nanjing, China). The activities of LDH (EC 1.1.1.28), SDH (EC 1.3.5.1), and AKP (EC 3.1.3.1) were measured according to the methods [64–66].

### Library construction and Illumina sequencing

Six cDNA libraries were constructed from *R. philippinarum* in the hypoxia challenged group (H1, H2 and H3) and control group (N1, N2 and N3) for RNA-seq analysis. The six RNA-seq libraries were performed at Novogene Co., Ltd. (Beijing, China) with a NEBNext Ultra RNA Library Prep Kit for Illumina (New England Biolabs, USA) following the manufacturer’s recommendations. mRNA was purified from total RNA using poly-T oligo-attached magnetic beads First strand cDNA was synthesized using random hexamer primers and M-MLV Reverse Transcriptase (RNaseH-). Second strand cDNA synthesis was subsequently performed using the DNA Polymerase I and RNase H. DNA fragments were treated for end-repairing, adenylation of 3′ ends and ligation of adaptors. The library fragments were purified with AMPure XP system (Beckman Coulter, CA, USA) to preferentially select cDNA fragments of 150 ~ 200 bp in length, and suitable fragments were enriched by PCR amplification. The library quality was assessed on the Bioanalyzer 2100 system (Agilent, USA). At last, the libraries were sequenced on an Illumina Hiseq platform and 150 bp paired-end reads were generated.

### Processing of raw reads, gene annotation and gene expression levels

Raw data (raw reads) of the fastQ format were firstly processed for quality trimming through in-house Perl Scripts. In this step, clean data (clean reads) were obtained by removing reads containing adapter, reads containing poly-N and low quality reads from raw data. All the downstream analyses were based on clean data with high quality. The clustering of the index-coded samples was performed on a cBot Cluster Generation System using Truseq PE Cluster Kit v3-cBot-HS (Illumia) according to the manufacture’s instructions. After cluster generation, the library preparation were sequenced on an Illumina Hiseq platform and 2 × 125 bp paired-end reads were generated. Clean sequences were deposited to NCBI Short Read Archive (SRA) database (http://www.ncbi.nlm.nih.gov/Traces/sra/). Clean data (clean reads) were obtained by removing the reads containing adaptors, reads with more than 10% poly-N and reads of low quality (the number of bases with sQ < = 5 accounts for more than 50% of the total read length) from the raw data. The remaining high quality clean reads were assembled using Trinity for transcriptome assembly without reference genome [67]. The longest transcript of each single gene was selected as a unigene. For annotation analysis, unigenes were BLASTX-searched against seven databases, including the National Center for Biotechnology Information (NCBI) non-redundant protein sequence (Nr) database, non-redundant nucleotide sequence (Nt) databases, Protein family (Pfam), Clusters of Orthologous Groups (KOG/COG), Gene Ontology (GO), Kyoto Encyclopedia of Genes and Genomes (KEGG) Orthology (KO) database, and the Swiss-prot, using a cut-off E-value of 10^− 5^. Differentially expressed genes (DEGs) between hypoxia challenged clam and untreated clam were identified with DEGseq analysis on adjusted read count data. To identify unigenes involved in Manila clam under hypoxia stress, pairwise comparisons for differential expression analysis were conducted among hypoxia treatment and its control group. Unigenes were annotated based on BLASTX results, and the best alignments were used for downstream analyses. KEGG database were used to predict the functions of unigenes.

Differentially expressed genes (DEGs) were measured by counting tags from hypoxia/thermal treated samples against the control, which are normalized using the RNA Sequence Expected Maximization (RSEM) method [68]. Initially, reads from control sample were mapped to reference transcriptome and subjected to check the differential expression using trinity utility scripts (align_and_estimate_abundance.pland abundance_estimates_to_matrix.pl) as instructed (http://trinityrnaseq.github.io/). The edgeR program package was applied and adjusted the read counts for each sequenced library using before the differential gene expression analysis [69]. To elucidate the differences between the hypoxia exposure and normoxia transcriptomes, DEGseq was used to screen the differentially expressed genes in hypoxia challenged group transcriptome library (three replicate: H1, H2 and H3) and the control group transcriptome library (three replicate: N1, N2 and N3) [70]. *P* < 0.05 and |log2 (fold change)| > 1 was set as the threshold for significantly DEGs.

### Experimental validation of transcription levels

To corroborate the RNA-seq results, we select both upregulated and downregulated hypoxia-responsive differentially expressed genes to perform the quantitative real-time PCR (qPCR) analysis. Different time points of hypoxia challenged clams were further performed and tested for the differentially expressed genes in different time point which indentified by RNA-seq analysis. The expressions of fourteen hypoxia responsive genes in gills of *R. philippinarum* were conducted at different time 0, 2, 5, and 8 d in hypoxia challenge group (DO: 2.0 mg/L) and control group (DO: 7.0 mg/L), respectively (three biological replicate for each gene and time point). The Primer 5.0 software was used to design primers (Premier Biosoft International, Inc.) (Additional file 5). RNA (about 500 ng) samples were measured and treated with RQ1 RNase-Free DNase (Promega) to remove genomic DNA. The β-actin was used as the internal control for the qPCR analysis. The cDNA was synthesized using a reverse transcriptase reagent kit (PrimeScript™ RT reagent Kit, Takara). The qPCR was performed with SYBR Premix Ex Taq II (Takara). The reactions were carried out in a total volume of 25 μl containing 2.5 μl of diluted cDNA, 2.5 μl of each primer, and 12.5 μl of SYBR Green PCR Master Mix, with the following cycling profile: 95 °C for 15 min for polymerase activation, followed by 40 cycles at 95 °C for 15 s, at 55 °C for 30 s, and at 70 °C for 30 s. Each sample was processed in triplicate in the Roche LightCycler 480 Real-Time PCR System (Roche). All data were analyzed using the 2 ^–△△Ct^ method [71].

Significant differences among sample data were analyzed using SPSS 20.0 for Windows (IBM SPSS Inc., Chicago, IL, USA) software package. A one-way analysis of variance (ANOVA) was used to compare the effect of hypoxia on gene expression in gills of Manila clam. A one-way ANOVA was used to compare the effects of hypoxia on three enzymes activities including AKP, SDH, and LDH activities, followed by Duncan’s multiple comparison tests. Differences were considered to be significant at *P* < 0.05.

## Supplementary information

**Additional file 1. **Gene Ontology (GO) categorization of the unigenes in cellular component, biological process, and molecular function from transcriptome of *R. philippinarum*.

**Additional file 2.** The graphical display of GO enrichment results with candidate targeted genes by directed acyclic graph (DAG). The color depth represents the enrichment degree. The branch represents the relationship of GO, which illustrate the scope from increasingly small from top to bottom.

**Additional file 3. **Kyoto Encyclopedia of Genes and Genomes (KEGG) assignment of unigenes in transcriptome of *R. philippinarum* under hypoxia stress.

**Additional file 4.** 75 differentially expressed unigenes, including 43 upregulated and 32 downregulated unigenes indentified between hypoxia stress group and normoxia control group (qvalue< 0.005 and |log2(foldchange)| > 1).

**Additional file 5.** Primers used in qPCR validation of hypoxia responsive genes identified by RNA-seq analysis.

## Data Availability

The raw sequences for *R. philippinarum* have been deposited in the NCBI PRJNA478917 (https://www.ncbi.nlm.nih.gov/bioproject/PRJNA478917). The RNA-seq datasets are available in the NCBI Sequence Read Archive (SRA) with the accession numbers SRP151767, SRS3494254, SRX4332302, SRR7462276, SRS3494255, SRX4332301 and SRR7462277.

## Abbreviations

ROS: Reactive oxygen species
AKP: Alkaline phosphatase
SDH: Succinate dehydrogenase
LDH: Lactate dehydrogenase
NADH: Nicotinamide adenine dinucleotide
RNA-seq: RNA sequencing
DEGs: Differentially expressed genes
qPCR: Quantitative real-time PCR
ANOVA: A one-way analysis of variance
HIF-1: Hypoxia-inducible factor 1
NF-κB: Nuclear factor kappa B
TNF: Tumor Necrosis Factor
HSP70: Heat shock protein 70
PEX: Peroxisomal membrane protein
SCP: Sterol carrier protein
GPx: Glutathione peroxidase
IAP: Inhibitor of apoptosis protein
