# Supplementary figures and images for: Transcriptome analysis reveals differential immune related genes expression in Ruditapes philippinarum under hypoxia stress: potential HIF and NF-κB crosstalk in immune responses in clam
Source: BMC Genomics. 2020 Apr 23;21:318. doi: 10.1186/s12864-020-6734-6 (PMC7181582; doi:10.1186/s12864-020-6734-6)

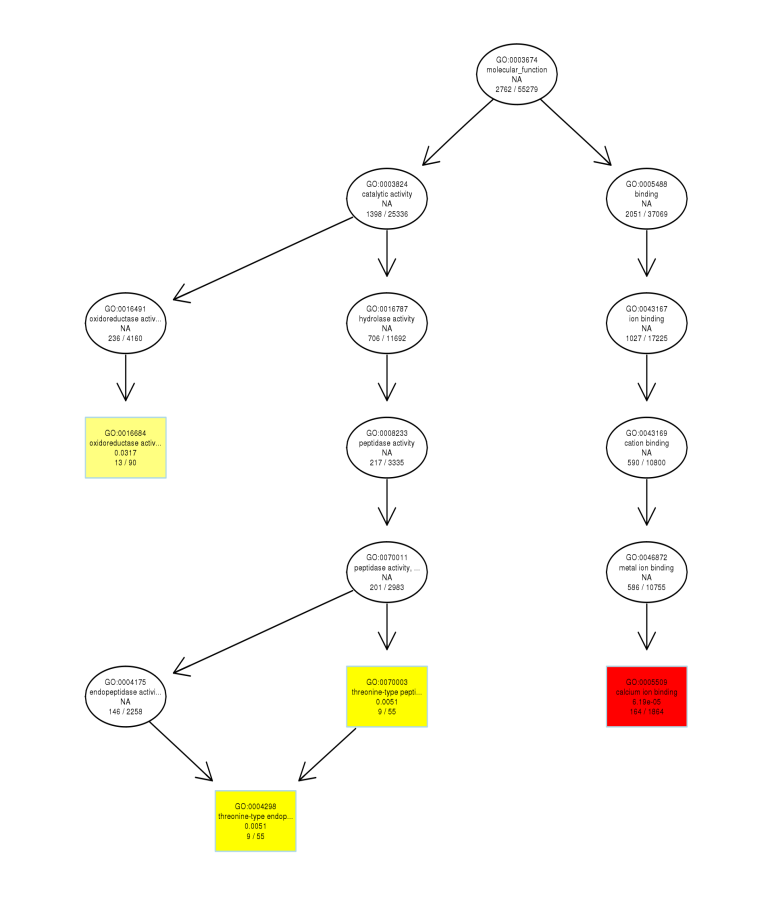

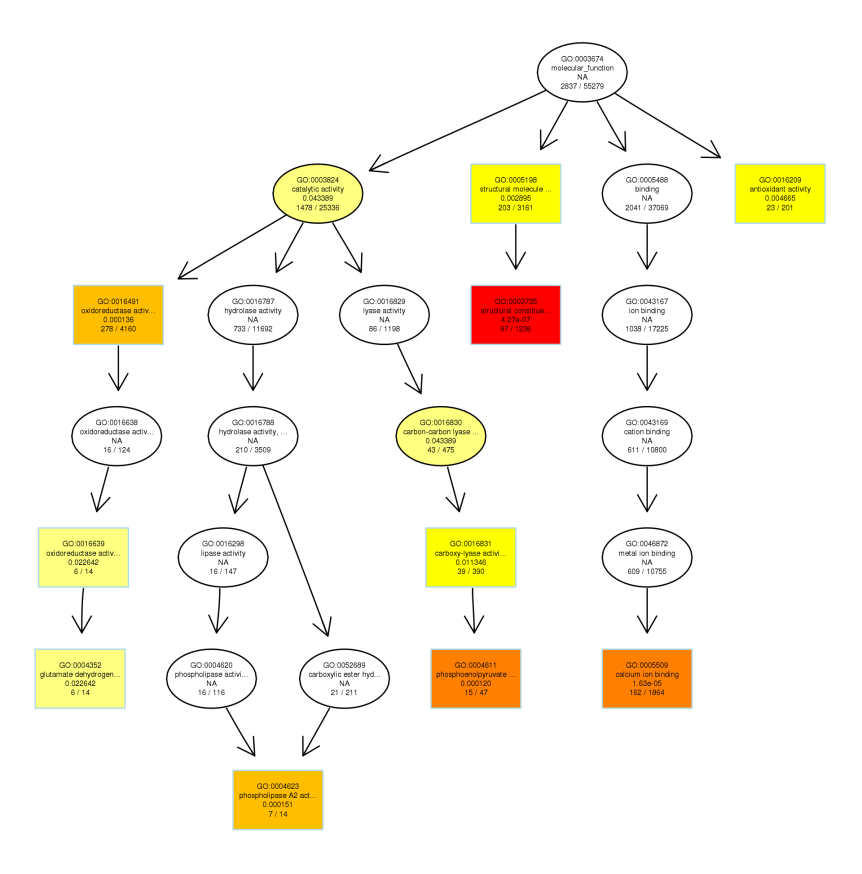

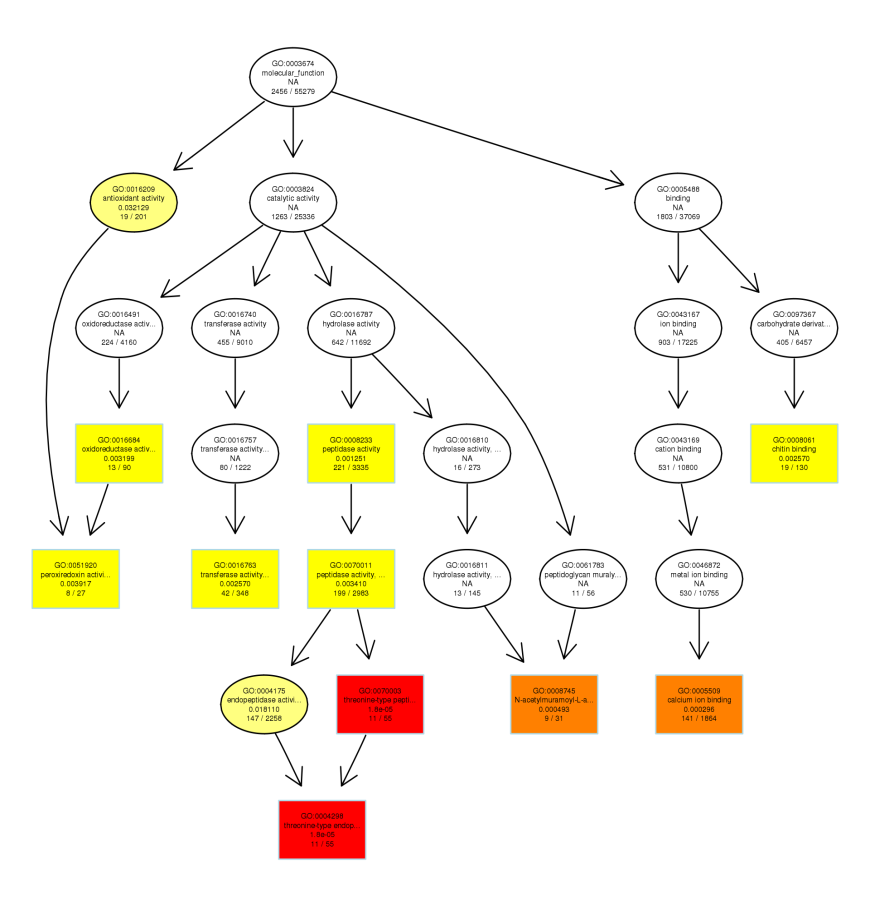

Supplement: Supplementary file 2 — Additional file 2. The graphical display of GO enrichment results with candidate targeted genes by directed acyclic graph (DAG). The color depth represents the enrichment degree. The branch represents the relationship of GO, which illustrate the scope from increasingly small from top to bottom. [file 12864_2020_6734_MOESM2_ESM.docx]
