# Supplementary material for: Transcriptome analysis reveals differential immune related genes expression in Ruditapes philippinarum under hypoxia stress: potential HIF and NF-κB crosstalk in immune responses in clam
Source: BMC Genomics. 2020 Apr 23;21:318. doi: 10.1186/s12864-020-6734-6 (PMC7181582; doi:10.1186/s12864-020-6734-6)
Supplement: Supplementary file 5 — Additional file 5. Primers used in qPCR validation of hypoxia responsive genes identified by RNA-seq analysis. [file 12864_2020_6734_MOESM5_ESM.docx]

**Additional file 5** Primers used in RT-qPCR assays.

| Gene | Gene ID | Oligonucleotide | Sequence | Tm (℃) |
| --- | --- | --- | --- | --- |
| HSP70 | Cluster-49988.108598 | Forward primer | ACAGTTATGGTAGGTGAGCCGC | 60 |
|  |  | Reverse primer | CTACTCGCCCAAGGTATTCCG | 60 |
| Cytochrome P450 | Cluster-49988.181088 | Forward primer | AGGGAGATACAGGGAGGACAGAC | 60 |
|  |  | Reverse primer | GGCATTTCCATAACCACATCG | 60 |
| Peroxisomal membrane protein PEX | Cluster-49988.141620 | Forward primer | CAAAGTGTTGGGTAAAAGACGAGG | 60 |
|  |  | Reverse primer | GCTTCGTTAGGAAATTCTCTGCTC | 60 |
| Sterol carrier protein SCP | Cluster-49988.126154 | Forward primer | CATCCACTTGGTGCTACAGGTCTA | 60 |
|  |  | Reverse primer | CGCACCCTTAACCTGTCTCTTG | 60 |
| Glutathione peroxidase GPx | Cluster-49988.129273 | Forward primer | GATTGGATAGGAAAGGACGGATG | 60 |
|  |  | Reverse primer | CACATGACAATGCAATCAGCTTG | 60 |
| Fibropellin-1 | Cluster-49988.124853 | Forward primer | GCATGCACTGTTGAACCTTGTC | 60 |
|  |  | Reverse primer | ACTCTATCTTCACACGATCCTCCG | 60 |
| Inhibitor of apoptosis protein IAP | Cluster-49988.128004 | Forward primer | AACACGGCAGAGAATAGGTCCA | 60 |
|  |  | Reverse primer | GCTGTTGTGATGTTGATGGGGT | 60 |
| Serine/threonine-protein phosphatase | Cluster-49988.116834 | Forward primer | CTCACACTGAAGGCAAAATGGC | 60 |
|  |  | Reverse primer | CAACAATATCTCCAGGCGTTGG | 60 |
| Ras-related protein | Cluster-49988.135730 | Forward primer | ATTGACCAGCCAAAACGTGAAG | 60 |
|  |  | Reverse primer | TTCCCAGAGACCAGGAGTTTGT | 60 |
| Cadherin | Cluster-49988.135471 | Forward primer | ATTTATCCTGAATGATCCCCCG | 60 |
|  |  | Reverse primer | GCCGACCTCAAACTCTTCACTG | 60 |
| Ubiquitin-conjugating enzyme E2 | Cluster-49988.130953 | Forward primer | GGACACAACACACCAGCGACA | 60 |
|  |  | Reverse primer | TCCGATATGAGGACACGCTTTC | 60 |
| Calmodulin | Cluster-49988.140627 | Forward primer | ATAGCCCTTAGTCCAACCAATGAG | 60 |
|  |  | Reverse primer | GGCTTTTGTGGCAATTATTGTC | 60 |
| Defensin | Cluster-49988.135418 | Forward primer | CAACAGGCTGAGCACTTGAC | 60 |
|  |  | Reverse primer | CGAATAGTCATTGGGGCAGC | 60 |
| E3 ubiquitin-protein ligase | Cluster-49988.140339 | Forward primer | CATCACCTATGAACTGACAACGGAC | 60 |
|  |  | Reverse primer | TGTCTTTCCACATCCTGTTTCACC | 60 |
